# TGF-β: Many Paths to CD103^+^ CD8 T Cell Residency

**DOI:** 10.3390/cells10050989

**Published:** 2021-04-23

**Authors:** Zhijuan Qiu, Timothy H. Chu, Brian S. Sheridan

**Affiliations:** Department of Microbiology and Immunology, Center for Infectious Diseases, Renaissance School of Medicine, Stony Brook University, Stony Brook, NY 11794, USA; zhijuan.qiu@stonybrook.edu (Z.Q.); timothy.chu@stonybrook.edu (T.H.C.)

**Keywords:** CD8 tissue-resident memory T cells, TGF-β, CD103, integrin, short-lived effector T cells, memory precursor effector T cells, Kruppel-like factor 2

## Abstract

CD8 tissue-resident memory T (T_RM_) cells primarily reside in nonlymphoid tissues without recirculating and provide front-line protective immunity against infections and cancers. CD8 T_RM_ cells can be generally divided into CD69^+^ CD103^−^ T_RM_ cells (referred to as CD103^−^ T_RM_ cells) and CD69^+^ CD103^+^ T_RM_ cells (referred to as CD103^+^ T_RM_ cells). TGF-β plays a critical role in the development and maintenance of CD103^+^ CD8 T_RM_ cells. In this review, we summarize the current understanding of tissue-specific activation of TGF-β mediated by integrins and how it contributes to CD103^+^ CD8 T_RM_ cell development and maintenance. Furthermore, we discuss the underlying mechanisms utilized by TGF-β to regulate the development and maintenance of CD103^+^ CD8 T_RM_ cells. Overall, this review highlights the importance of TGF-β in regulating this unique subset of memory CD8 T cells that may shed light on improving vaccine design to target this population.

## 1. Introduction

Immunological memory is a hallmark of adaptive immunity and the basis of vaccination. Memory CD8 T cells, as an important component of immunological memory, play a critical role in mediating long-term protective immunity against intracellular pathogens and cancers. Based on their migratory patterns, memory CD8 T cells can be divided into circulating effector memory T (T_EM_) and central memory T (T_CM_) cells and noncirculating tissue-resident memory T (T_RM_) cells [1,2,3,4,5,6,7]. T_EM_ cells recirculate between blood, nonlymphoid tissues, and secondary lymphoid organs. T_CM_ cells recirculate between blood and secondary lymphoid organs. T_RM_ cells reside within a particular tissue and do not recirculate during homeostasis. Circulating memory T cells and T_RM_ cells differ in their protective nature and timing for immunologic impact. Upon re-exposure to pathogens, while circulating memory T cells need to be recruited to infected tissues to exert their protective function, T_RM_ cells are prepositioned in the tissue to respond immediately, representing a first-line defense against invading pathogens. TGF-β is a pleiotropic cytokine that plays an important role in regulating many cellular processes such as fibrogenesis and carcinogenesis [8,9,10,11]. It is produced and sensed by a variety of immune cells and regulates many aspects of an immune response [11,12,13]. While the role of TGF-β in the formation of CD103^+^ CD8 T_RM_ cells appears absolute and is the focus of this review, TGF-β signaling is also important in numerous other immunologic processes associated with CD8 T cells, such as the formation and maintenance of circulating memory CD8 T cells [14]. TGF-β signals provided during the effector phase favors memory precursor effector cells and promotes the formation of circulating memory CD8 T cells [14,15,16], while TGF-β signaling provided during memory homeostasis controls the maintenance of circulating memory CD8 T cells by preserving their phenotypic and functional identity [14]. This review focuses on the diverse role of TGF-β in the development and maintenance of CD103^+^ CD8 T_RM_ cells by skewing the population towards a memory precursor effector cell (MPEC) phenotype and by regulating *Itgae* (which encodes CD103) and *Klf2* expression.

## 2. CD8 T_RM_ Cells and Their Protective Functions

CD8 T_RM_ cells primarily reside in nonlymphoid tissues and are phenotypically, functionally, transcriptionally, and metabolically distinct from circulating memory T cell subsets [1,2,3,17,18,19,20,21,22,23,24,25,26]. Phenotypically, CD8 T_RM_ cells predominately express CD69, an albeit imperfect marker that is commonly used to distinguish CD8 T_RM_ cells from their circulating counterparts, and the majority of those CD8 T_RM_ cells that reside within epithelium also express the α_E_ integrin, CD103 [2,27,28]. CD69 interacts with sphingosine-1-phosphate receptor S1PR1 and promotes early CD8 T cell retention in nonlymphoid tissues by inhibiting S1PR1-mediated tissue egress [18,29,30,31,32,33]. CD103 binds to E-cadherin expressed in adherens junctions of epithelial cells and plays an important role in the accumulation and retention of CD8 T_RM_ cells in barrier tissues [18,27,31,34,35]. CD8 T_RM_ cells functionally differ from circulating memory T cells; however, the difference varies among tissues and subjects of studies. For example, CD8 T_RM_ cells in the small intestine epithelium express more granzyme B but less cytokines IFNγ, TNFα, and IL-2 than circulating memory T cells [17,27]. While CD8 T_RM_ cells in the mouse liver express more granzyme B, IFNγ, and TNFα than circulating memory T cells [36], those in the human liver display lower granzyme B, higher perforin, IFNγ, and IL-2 than circulating memory T cells [37]. CD8 T_RM_ cells in the skin express higher CD49a than their circulating counterparts, a marker that is associated with the expression of cytotoxic effector proteins such as granzyme B and perforin [2,38], suggesting that skin CD8 T_RM_ cells may be more cytotoxic than circulating memory T cells. CD8 T_RM_ cells exhibit a unique transcriptional profile that is fundamentally distinct from circulating memory T cells [18,19,20,21,22,23]. A core gene signature comprising both upregulated and downregulated genes shared by CD8 T_RM_ cells at different anatomic locations, including the skin, lung, and small intestine, has been defined [18]. Among upregulated genes are various adhesion molecules like *Itga1* (encodes CD49a), *Itgae* (encodes CD103), and *Cdh1* (encodes E-cadherin) that are known or likely to be associated with tissue retention, while the most prominent downregulated gene is *S1pr1* that is required for tissue egress, all of which presumably contribute to the establishment and maintenance of tissue residency. Finally, CD8 T_RM_ cells are metabolically distinct from circulating memory T cells. CD8 T_RM_ cells, but not their circulating memory counterparts, upregulate fatty-acid-binding protein (FABP) 4 and 5 expression, allowing them to use exogenous free fatty acid and oxidative metabolism for survival and long-term maintenance in the tissue [26]. A recent study showed that tumor cells also upregulate FABP4 and FABP5 expression and outcompete tumor reactive CD8 T_RM_ cells for lipid uptake leading to T_RM_ cell death [39]. Interestingly, PD-L1 blockade decreases FABP4 and FABP5 expression in tumor cells while upregulating expression of these molecules in T_RM_ cells, promoting T_RM_ cell survival and their antitumor response [39].

CD8 T_RM_ cells play a critical role in protective immunity against infections and cancers [2,7,25,34,36,40,41,42,43,44,45,46]. They are prepositioned in the tissue to respond immediately to pathogen re-encounter and mediate protective immunity through multiple mechanisms. CD8 T_RM_ cells express high levels of cytotoxic granules such as granzyme B and can rapidly and directly lyse infected cells [17,27,36,38]. Upon reactivation by local antigen, CD8 T_RM_ cells rapidly release cytokines including IFNγ, TNFα, and IL-2. IFNγ in turn triggers tissue-wide expression of diverse antimicrobial genes [47], and induces expression of vascular cell adhesion molecule 1 (VCAM-1) on local endothelial cells, which mediates the recruitment of circulating memory T and B cells [48,49]. TNFα is essential for the maturation of dendritic cells by promoting the lymph node homing receptor CCR7 and costimulatory molecule CD86 expression, while IL-2 is critical for NK cell activation by inducing granzyme B expression [49]. Upon antigenic rechallenge, CD8 T_RM_ cells undergo profound in situ proliferation, contributing to the local recall response and secondary T_RM_ cell populations [50,51]. Finally, antigenic reactivation can lead to the lymphatic exit of CD8 T_RM_ cells from their tissue of residence to rejoin the circulation [52,53,54]. These former CD8 T_RM_ cells have the plasticity to give rise to circulating memory T cells, preferably circulating effector memory T cells, at different anatomical locations, as well as T_RM_ cells in the secondary lymphoid organs [52,53,54]. They also maintain the propensity to return to their tissue of origin and redifferentiate into T_RM_ cells [53,54]. Of note, a recent study showed that aged mice exhibit excessive accumulation of CD8 T_RM_ cells in the lung after a viral infection as a result of elevated TGF-β in the infected aged lung [55]. These CD8 T_RM_ cells display diminished effector functions and consequently fail to provide protective immunity against secondary infection. Instead, they cause chronic lung inflammation and fibrosis. Therefore, CD8 T_RM_ cells that provide protective immunity in young individuals may be detrimental in aged individuals.

## 3. The Role of TGF-β in CD8 T_RM_ Cell Development and Maintenance

Studies in the past decade have demonstrated that TGF-β signaling is required for the development and maintenance of CD103^+^ CD8 T_RM_ cells, but not CD103^−^ CD8 T_RM_ cells, in many tissues including the lung, small intestine, skin, salivary glands, and brain [18,27,31,34,35,56,57,58,59,60]. Of note, TGF-β signaling promotes the accumulation of CD103^−^ CD8 T_RM_ cells in the extravascular compartment of the kidney by promoting effector T cell extravasation [61]. TGF-β signaling does not seem to affect the overall accumulation of effector CD8 T cells in the tissue at the peak of the T cell response [18,34,57]. However, in the absence of TGF-β signaling, tissue-infiltrating CD8 T cells are not able to upregulate CD103 expression and fail to differentiate into CD103^+^ CD8 T_RM_ cells [18,27,31,34,35,57]. Moreover, tissue-infiltrating cells that fail to receive TGF-β signals are not maintained long term in the tissue [18,34,57]. Interestingly, during chronic lymphocytic choriomeningitis virus infection, although CD8 T cells that lack TGF-β signaling are not able to differentiate into CD103^+^ CD8 T_RM_ cells, they are maintained efficiently in the tissue due to the continuous replenishment of effector CD8 T cells from the circulation [57]. In this model, CD8 T cells that lack TGF-β signaling have increased gut-homing receptor α_4_β_7_ expression and an enhanced ability to migrate to the tissue in the presence of continued antigen and/or inflammation [57], though this was not observed in a model of a rapidly controlled bacterial infection [34].

## 4. Integrin-Mediated Activation of TGF-β in the Development and Maintenance of CD103^+^ CD8 T_RM_ Cells

**Figure 1:**
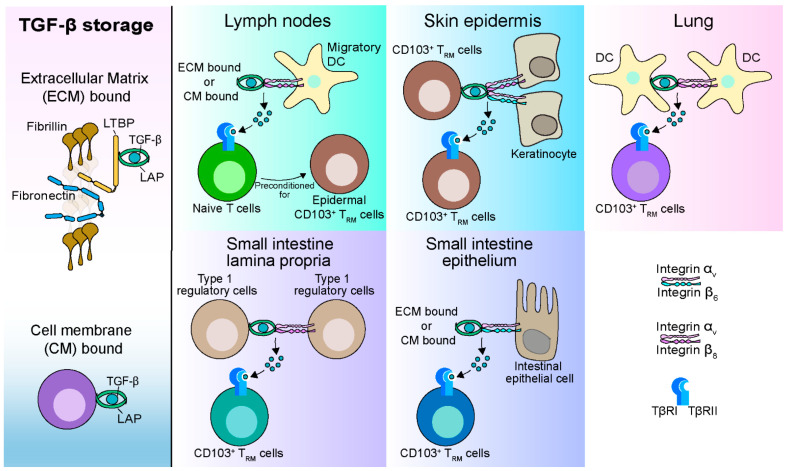
Integrin-mediated activation of TGF-β in the development and maintenance of CD103^+^ CD8 T_RM_ cells. TGF-β is secreted as LAP-TGF-β latent complex, which binds to the latent TGF-β binding protein (LTBP) to form the large latent complex. The large latent complex can bind to extracellular matrix (ECM) proteins such as fibronectin and fibrillin to facilitate the deposit and storage of TGF-β into the ECM. Alternatively, LAP-TGF-β latent complex can also be deposited and stored on the surface of cell membranes. The activation of TGF-β requires the release of TGF-β from the LAP-TGF-β latent complex. The best understood mechanism is through integrin α_v_β_8_ and α_v_β_6_. During homeostasis in the lymph nodes, α_v_β_8_ expressed by migratory dendritic cells (DC) activates and presents TGF-β to precondition native CD8 T cells for epidermal CD103^+^ CD8 T_RM_ cell residency. In the skin epidermis, α_v_β_8_ and α_v_β_6_ expressed by different keratinocytes activate CD8 T_RM_ cell-derived TGF-β that is critical for the development and maintenance of CD103^+^ CD8 T_RM_ cells. In the small intestine lamina propria, type 1 regulatory cells promote CD103^+^ CD8 T_RM_ cell development by producing TGF-β and activating it through the expression of α_v_β_8_. In the small intestine epithelium, intestinal epithelial cells express α_v_β_6_, which is required for the activation of TGF-β and CD103^+^ CD8 T_RM_ cell development and maintenance. In the lung, DC membrane bound TGF-β and DC expressing α_v_β_8_ efficiently drive CD103^+^ CD8 T_RM_ cell differentiation.

TGF-β is synthesized as a precursor, consisting of the N-terminal latency-associated peptide (LAP) and the C-terminal mature cytokine [11,12,13,62]. The LAP is cleaved from the mature TGF-β cytokine in the Golgi by protease furin but remains noncovalently associated with the cytokine, preventing the access of the cytokine to its receptor. The LAP-TGF-β complex (also referred to as the small latent complex, SLC) can further associate with latent TGF-β binding protein (LTBP) through interactions with LAP to form the large latent complex (LLC). The LTBP can bind to proteins of the extracellular matrix (ECM) such as fibronectin and fibrillin and facilitate the deposition and storage of the LLC into the ECM after secretion. The activation of TGF-β requires the release of active TGF-β from the latent complex (Figure 1). The most effective and understood mechanism to activate TGF-β is through the integrins α_v_β_6_ and α_v_β_8_, which bind to the arginine–glycine–aspartic acid (RGD) motif in the LAP causing the release of TGF-β from the latent complex though physical force [11,12,13,62].

Integrin-mediated TGF-β activation plays an important role in multiple steps of CD103^+^ CD8 T_RM_ cell development and their long-term maintenance. During homeostasis, α_v_-expressing migratory dendritic cells (DC) activate TGF-β and present it to naïve CD8 T cells in lymph nodes through noncognate but MHC I-dependent interaction to precondition these naïve CD8 T cells for effective differentiation into epidermal CD103^+^ CD8 T_RM_ cells in the skin upon immune activation [63]. While resident DC expressing α_v_ integrins in the epidermis do not seem to play a role in the development of epidermal CD103^+^ CD8 T_RM_ cells, keratinocytes expressing α_v_β_6_ and α_v_β_8_ are required for the development and maintenance of CD103^+^ CD8 T_RM_ cells in the epidermis [63,64,65]. Several cell types in the epidermis, including Langerhans cells, keratinocytes, dendritic epidermal T cells (DETC), and CD8 T_RM_ cells, can express TGF-β and provide a source to support CD103^+^ CD8 T_RM_ cell residence [66]. Nevertheless, continued exposure to TGF-β derived from CD8 T_RM_ cells, but not Langerhans cells, keratinocytes, or DETC, is required for the long-term persistence of epidermal CD103^+^ CD8 T_RM_ cells [64,67,68]. Interestingly, while the presence of antigen in the flank skin is not required for the development of CD103^+^ CD8 T_RM_ cells, it renders antigen-specific T_RM_ cells more resistant to the impact of limited amounts of TGF-β than recruited bystander T_RM_ cells [68,69]. Therefore, TGF-β provides a competitive pressure that promotes the persistence of antigen-specific CD8 T_RM_ cells in the skin epidermis. However, how integrin-mediated TGF-β activation regulates CD103^+^ CD8 T_RM_ cell development and maintenance in the dermis is unclear.

In the small intestine, intestinal epithelial cells express α_v_β_6_ but not α_v_β_8_, and α_v_β_6_ is required for the development and maintenance of CD103^+^ CD8 T_RM_ cells in the intestinal epithelium but not lamina propria (LP) [64]. However, the source of TGF-β that supports CD103^+^ CD8 T_RM_ cell development and maintenance in the epithelium is unclear. Interestingly, in the LP, T-bet expressing type 1 regulatory cells are recruited to the LP through chemokine receptor CXCR3 and promote CD103^+^ CD8 T_RM_ cell development in the LP by producing TGF-β and activating it through the expression of α_v_β_8_ [70]. Therefore, the development and maintenance of CD103^+^ CD8 T_RM_ cells in the intestinal epithelium and LP require different α_v_ integrins and integrin-expressing cells.

In the lung, DC, in particular CD103^+^ DC, express α_v_β_8_ and are proficient at producing biologically active TGF-β to efficiently drive CD103 expression on CD8 T cells [58]. Studies using humanized mice demonstrated that human lung CD1c^+^ DC (which corresponds closely to murine cDC2) promote CD8 T cell accumulation in lung epithelia and present membrane-bound TGF-β to drive CD103^+^ T_RM_ cell differentiation [71]. Both studies identified the ability of DC to present TGF-β to induce CD103 expression and the differentiation of CD103^+^ CD8 T_RM_ cells, although the responsible DC subset differs between studies. However, whether TGF-β derived from other sources such as lung epithelial cells and other integrin-expressing cells play a role in CD103^+^ CD8 T_RM_ cell development in the lung needs further investigation.

## 5. Mechanisms by Which TGF-β Regulates the Development and Maintenance of CD103^+^ CD8 T_RM_ Cells

**Figure 2:**
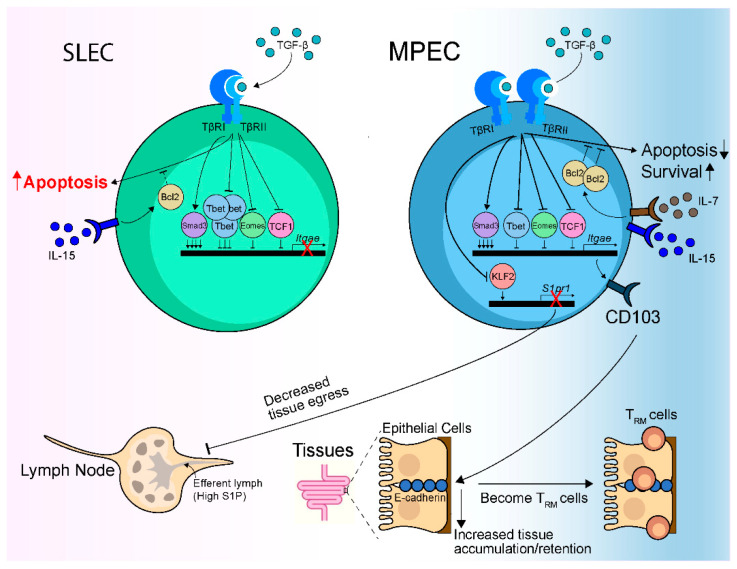
TGF-β regulates the development and maintenance of CD103^+^ CD8 T_RM_ cells through multiple mechanisms. (1) By skewing towards an MPEC phenotype—MPEC, but not SLEC, can form CD103^+^ CD8 T_RM_ cells. The survival of SLEC depends on IL-15, which cannot overcome TGF-β-mediated apoptosis. MPEC can respond to both IL-15 and IL-7, and IL-7 is able overcome the apoptotic effect induced by TGF-β. As a result, TGF-β selectively promotes the apoptosis of SLEC, leading to the accumulation of MPEC that favors the development of CD103^+^ CD8 T_RM_ cells. (2) Through regulating CD103 expression—TGF-β can directly induce CD103 expression through Smad3 or indirectly promote CD103 expression by counteracting T-bet, Eomes, and TCF1-mediated suppression of CD103 expression, thus supporting CD103^+^ CD8 T_RM_ cell development and maintenance through CD103-mediated tissue accumulation and retention. Although both SLEC and MPEC can respond to TGF-β, SLEC do not appear to express CD103 likely due to their lower TGF-βRII expression and the enhanced suppression of *Itgae* (the gene encoding CD103) expression by their heightened T-bet expression. (3) Through regulating KLF2 expression—TGF-β can downregulate KLF2 expression, which in turn downregulates S1PR1 expression and prevents S1PR1-mediated tissue egress of CD8 T cells, thus promoting the development of CD8 T_RM_ cells in the tissue.

Integrin-activating TGF-β is required for the development and maintenance of CD103^+^ CD8 T_RM_ cells in several tissues. Although it is incompletely understood, TGF-β can regulate CD103^+^ CD8 T_RM_ cell development and maintenance through multiple mechanisms (Figure 2).

### 5.1. By Skewing towards an MPEC Phenotype

TGF-β, whose production is increased after acute infection [15,72], selectively promotes the apoptosis of short-lived effector T cells (SLEC, CD127^−^ KLRG-1^+^) during clonal expansion and contraction by dampening antiapoptotic molecule B-cell lymphoma (Bcl)-2 expression and indirectly promoting the establishment of a predominately memory precursor effector T cells (MPEC, CD127^+^ KLRG-1^−^) population in lymphoid tissues over time [15]. TGF-β has also been shown to directly downregulate KLRG-1 expression [73], possibly further contributing to a decreased SLEC population. Indeed, in the absence of TGF-β signaling, a higher percentage of SLEC, and a lower percentage of MPEC were observed [15,34]. Previous studies have demonstrated that early effector cells and MPEC, but not SLEC, can form CD103^+^ CD8 T_RM_ cells [18,34]. Therefore, TGF-β can support CD103^+^ CD8 T_RM_ cell development by promoting SLEC apoptosis and skewing the population towards an MPEC phenotype.

MPEC and SLEC in lymphoid tissues express similar gut-homing receptor α_4_β_7_ expression and can both initially seed the small intestine [34]. However, MPEC rapidly accumulate in the small intestine in association with a rapid loss of SLEC that is primarily due to the accelerated apoptosis of SLEC [34]. This rapid shift to MPEC in the intestine occurs while the corresponding antigenic-specific splenic cells are largely SLEC, suggesting the differentiation pattern can be greatly influenced by the tissue-specific environment [34,74]. SLEC do not express CD127, and their survival is predominantly provided by IL-15, while MPEC express CD127 and their survival can be promoted by both IL-7 and IL-15 [75]. Both IL-7 and IL-15 can induce the antiapoptotic molecule Bcl-2 expression and thus promote cell survival. MPEC express higher Bcl-2 than SLEC, possibly due to their ability to respond to both IL-7 and IL-15 [15]. However, IL-7 but not IL-15 seems to be able to overcome the apoptotic effect induced by TGF-β [15]. TGF-β is constitutively expressed in the small intestine [76]. Therefore, after seeding the small intestine, SLEC undergo accelerated apoptosis in response to TGF-β in the tissue due to their inability to overcome TGF-β-mediated apoptosis, leading to the rapid accumulation of MPEC [34]. On the other hand, MPEC receive signals through TGF-β but do not appear to undergo enhanced apoptosis [34].

Naive T cells express high levels of TGF-β receptors, and the expression is transiently downregulated during T cell activation [15,77]. Activated T cells then regain the expression of TGF-β receptors and sensitivity to TGF-β. Extracellular ATP sensing through the purinergic receptor P2RX7 on CD8 T cells is required for the re-expression of TGF-β receptor II (TGF-βRII) [16]. The re-expression of TGF-βRII through P2RX7 potentiates the ability of CD8 T cells to respond to TGF-β, which promotes the MPEC population and is crucial for the generation of CD103^+^ CD8 T_RM_ cells [16].

### 5.2. Through Regulating CD103 Expression

It has long been known that TGF-β induces CD103 expression upon T cell activation in vitro [27,78,79]. Studies from the last decade demonstrated that TGF-β is absolutely required for CD103 upregulation and CD103^+^ CD8 T_RM_ cell differentiation in vivo [18,34,35,57,58]. CD103 plays a clear and critical role in CD103^+^ CD8 T_RM_ cell residence within the tissue. However, some studies suggest that CD103 is important for the long-term retention of CD103^+^ CD8 T_RM_ cells in the tissue [18,27,31,80], while others suggest that it promotes initial accumulation of effector CD8 T cells in the tissue [34,35]. CD103 can bind to E-cadherin expressed on epithelial cells and mediate heterotypic adhesive interaction between CD8 T cells and epithelial cells [81], likely contributing to the accumulation and retention of CD8 T cells in the tissue. However, concrete evidence demonstrating the contribution of CD103-E-cadherin interaction to effector CD8 T cell accumulation and CD103^+^ CD8 T_RM_ cell retention in the tissue is so far lacking. Recent studies demonstrated that CD103-deficient CD8 T_RM_ cells have increased motility [82,83], indirectly supporting that CD103 on CD103^+^ CD8 T_RM_ cells may interact with E-cadherin on epithelial cells to restrain the motility of T_RM_ cells and therefore enhance their retention. Furthermore, CD103^+^ CD8 T_RM_ cells have been shown in several studies to have higher expression of antiapoptotic molecule Bcl-2 than CD103^−^ CD8 T_RM_ cells [18,35,80], suggesting that CD103^+^ CD8 T_RM_ cells have a survival advantage over CD103^-^ CD8 T_RM_ cells. However, whether CD103 is involved in Bcl-2 expression or simply marks a subset of effector cells receiving IL-15 and/or IL-7 signals needs further clarification.

TGF-β binds to TGF-βRII, which then recruits and phosphorylates TGF-βRI, resulting in the activation of TGF-βRI [10,12]. Active TGF-βRI recruits and phosphorylates receptor-activated Smad (R-Smad) proteins Smad2 and Smad3, which can subsequently bind to the co-Smad protein Smad4 [10,12]. The Smad2/3/4 complex translocates to the nucleus, where it binds to Smad-binding elements in target genes and initiates their transcription [10,12]. TGF-β in the absence of T cell receptor (TCR) signaling activates downstream Smad2/3 but fails to induce CD103 expression. Similarly, TCR engagement in the absence of TGF-β signaling activates nuclear factor of activated T cells (NFAT) but does not induce CD103 expression [84]. TGF-β together with TCR engagement are needed to induce CD103 expression [84]. Smad3 activated by TGF-β and NFAT activated by TCR engagement bind to their respective responsive elements in the promoter and enhancer regions of *Itgae* gene (the gene that encodes CD103), together driving the expression of CD103 [84].

T-bet can directly bind to *Itgae* gene locus in virus-specific CD8 T cells to suppress CD103 expression and CD103^+^ CD8 T_RM_ cell formation, presumably through interfering with the binding of Smad3 to the *Itgae* gene locus [72]. Indeed, overexpression of T-bet in activated CD8 T cells inhibits CD103 expression and CD103^+^ CD8 T_RM_ cell formation [72,85]. Moreover, T-bet can depress TGF-βRII expression on activated CD8 T cells, which likely negatively regulates CD8 T cell responsiveness to TGF-β and the induction of CD103 [85]. Interestingly, TGF-β signaling through TGF-βRII can downregulate T-bet expression, which in turn mitigates the suppression effect of T-bet on CD103 expression [85]. Similar reciprocal regulation was also observed between TGF-β and another T-box transcription factor Eomesodermin (Eomes) [85]. T cell factor 1 (TCF1) can also directly bind to *Itgae* gene locus to suppress CD103 expression and CD103^+^ CD8 T_RM_ cell formation [86]. Interestingly, TGF-β can inhibit TCF1 protein expression and reduce the recruitment of TCF1 to the *Itgae* gene locus, which represses the negative regulation of TCF1 on CD103 expression [86]. TCF1 is critical for T_CM_ cell differentiation and longevity [87,88]. A recent study identified heterogenous populations of CD8 T_RM_ cells in the intestine that included a Blimp-1^hi^ Id3^lo^ cell population that decreases over time and a Blimp-1^lo^ Id3^hi^ cell population that increases over time [89]. Blimp-1^lo^ Id3^hi^ T_RM_ cells express higher TCF1, suggesting that TCF1 may promote their longevity. Paradoxically, Blimp-1^lo^ Id3^hi^ T_RM_ cells express higher CD103, contradictory to the known role of TCF1 in suppressing CD103 expression. During the effector phase, Blimp-1^lo^ Id3^hi^ cells resemble MPEC, while Blimp-1^hi^ Id3^lo^ cells resemble SLEC. As MPEC express higher TGF-βRII and lower T-bet than SLEC [15,90]; this suggests that Blimp-1^lo^ Id3^hi^ cells may have higher TGF-βRII and lower T-bet expression. Higher TGF-βRII expression may enhance CD103 expression, while lower T-bet expression may result in reduced suppression of CD103 expression. However, how TGF-β signaling, T-bet, and TCF1 regulate CD103 expression in these T_RM_ cell populations require further evaluation.

Overall, TGF-β regulates CD103 expression and CD103^+^ CD8 T_RM_ cell differentiation by directly driving CD103 expression on activated CD8 T cells through Smad3 and indirectly promoting CD103 expression through antagonizing T-bet, Eomes and TCF1-mediated suppression of CD103 expression.

Both SLEC and MPEC express TGF-βRII and can respond to TGF-β [15]. However, while MPEC quickly upregulate CD103 expression in response to TGF-β in the small intestine, SLEC fail to do so [34]. Previous studies demonstrated that SLEC express lower TGF-βRII and three- to four-fold more T-bet than MPEC [90]. As TGF-β signaling through TGF-βRII induces CD103 expression and T-bet can inhibit TGF-β-Smad3-mediated induction of CD103 expression [72], the lower TGF-βRII expression and higher T-bet expression in SLEC likely leads to a failure to upregulate CD103 expression. Therefore, SLEC cannot form CD103^+^ CD8 T_RM_ cells due to an inability to upregulate CD103 expression and an inability to survive in response to TGF-β.

While TGF-β and CD103 are intimately linked, CD103-deficient CD8 T cells do not mirror TGF-βRII-deficient CD8 T cells. For example, TGF-βRII deficiency leads to a severe defect (20- to 50-fold reduction) in the development and maintenance of CD8 T_RM_ cells in the intestinal epithelium, while CD103 deficiency only resulted in a mild defect (two- to three-fold reduction) [27,34,57]. Thus, TGF-β utilizes other pathways to promote CD8 T_RM_ cell development and maintenance in addition to regulating the expression of the prototypical marker of epithelial residency CD103.

### 5.3. Through Regulating KLF2 Expression

TGF-β alone or in combination with IL-33 and/or TNF can downregulate the zinc-finger transcription factor Kruppel-like factor 2 (KLF2) through a PI3K/Akt dependent pathway [32]. As KLF2 directly binds to the promoter of the gene encoding S1PR1 to induce S1PR1 expression, TGF-β signals lead to loss of S1PR1 expression [91,92]. S1PR1 mediates the egress of CD8 T cells from tissues by binding to its ligand S1P found at very high concentrations in the afferent lymphatics draining peripheral tissues. KLF2 and S1PR1 are critical in the induction of T_RM_ cells, as forced expression of KLF2 or S1PR1 prevents the establishment of CD8 T_RM_ cells [32]. Downregulation of S1PR1 expression at the gene expression level through the downregulation of KLF2 or at the surface protein expression level through the interaction with CD69 is essential for preventing tissue egress of CD8 T cells and the establishment of CD8 T_RM_ cells [32,33]. Therefore, TGF-β can further promote CD8 T_RM_ cell formation by downregulating KLF2 and S1PR1 expression to facilitate T cell retention in peripheral nonlymphoid tissues.

## 6. Conclusions and Implications

CD8 T_RM_ cells provide front-line protective immunity against infections and cancers. TGF-β plays a crucial role in regulating the development and maintenance of CD103^+^ CD8 T_RM_ cells through multiple mechanisms. TGF-β is secreted as a latent form that needs to be activated to exert its function. The activation of TGF-β is mediated by different α_v_ integrins and integrin-expressing cells at different tissues, highlighting tissue-specific regulation of TGF-β activation and CD103^+^ CD8 T_RM_ cell development and maintenance. TGF-β can induce the apoptosis of SLEC and promote the accumulation of MPEC that favors the development of CD103^+^ CD8 T_RM_ cells. TGF-β can directly induce CD103 expression or indirectly promote CD103 expression by counteracting mechanisms that suppress CD103 expression and thus support CD103^+^ CD8 T_RM_ cell development and maintenance through CD103-mediated tissue accumulation and retention. Furthermore, TGF-β can downregulate KLF2 expression, which in turn downregulates S1PR1 expression and prevents S1PR1-mediated tissue egress of CD8 T cells, thus promoting the development of CD8 T_RM_ cells in the tissue. However, whether there are additional TGF-β mediated mechanisms that promote the development and maintenance of CD103^+^ CD8 T_RM_ cells needs further investigation. Understanding exactly how TGF-β regulates the development and maintenance of CD103^+^ CD8 T_RM_ cells can help us better target this memory population for protection against infections and malignancies. For example, MPEC but not SLEC can overcome TGF-β-induced apoptosis and form CD103^+^ CD8 T_RM_ cells in response to TGF-β. Approaches that favor MPEC generation such as using the TLR4 ligand LPS as an adjuvant for DC vaccination may promote CD103^+^ CD8 T_RM_ cell development [93]. CD8 T_RM_ cells have also been implicated in autoimmune and inflammatory diseases, such as psoriasis [6]. Blocking TGF-β activation in the skin may provide a therapeutic approach to eliminate skin CD103^+^ CD8 T_RM_ cells with minimal impact on other immune compartments. Finally, a better understanding of the role of TGF-β in CD103^+^ CD8 T_RM_ cell development and maintenance may lead to improved antitumor immunotherapies. It is becoming increasingly evident that CD8 T_RM_ cells provide superior protection against tumors. However, it is well known that TGF-β is a critical factor that promotes tumor development and metastasis. Thus, understanding these pathways more comprehensively could lead to targeted therapies to promote antitumor CD8 T_RM_ cells.
